# LOADng-IoT: An Enhanced Routing Protocol for Internet of Things Applications over Low Power Networks

**DOI:** 10.3390/s19010150

**Published:** 2019-01-03

**Authors:** José V. V. Sobral, Joel J. P. C. Rodrigues, Ricardo A. L. Rabêlo, Kashif Saleem, Vasco Furtado

**Affiliations:** 1 Instituto de Telecomunicações, Universidade da Beira Interior, 6201-001 Covilhã, Portugal; jose.sobral@it.ubi.pt; 2 Department of Education, Federal Institute of Maranhão (IFMA), R. Afonso Pena, 174, São Luís-MA 65010-030, Brazil; 3 National Institute of Telecommunications (Inatel), Santa Rita do Sapucaí-MG 37540-000, Brazil; 4 Center of Excellence in Information Assurance (CoEIA), King Saud University, Riyadh 11653, Saudi Arabia; ksaleem@ksu.edu.sa; 5 Department of Computing, Federal University of Piauí (UFPI), Teresina-PI 64049-550, Brazil; ricardoalr@ufpi.edu.br; 6 Programa de Pos-Graduação em Informática Aplicada (PPGIA), University of Fortaleza (UNIFOR), Av. Washington Soares, 1321, Fortaleza-CE 60811-905, Brazil; vasco@unifor.br

**Keywords:** internet of things, LOADng, LOADng-IoT, low power and lossy networks, routing protocol

## Abstract

The Internet of Things (IoT) is an emerging paradigm that proposes the connection of objects to exchange information in order to reach a common objective. In IoT networks, it is expected that the nodes will exchange data between each other and with external Internet services. However, due to deployment costs, not all the network devices are able to communicate with the Internet directly. Thus, other network nodes should use Internet-connected nodes as a gateway to forward messages to Internet services. Considering the fact that main routing protocols for low-power networks are not able to reach suitable performance in the displayed IoT environment, this work presents an enhancement to the Lightweight On-demand Ad hoc Distance-vector Routing Protocol—Next Generation (LOADng) for IoT scenarios. The proposal, named LOADng-IoT, is based on three improvements that will allow the nodes to find Internet-connected nodes autonomously and dynamically, decreasing the control message overhead required for the route construction, and reducing the loss of data messages directed to the Internet. Based on the performed assessment study, which considered several number of nodes in dense, sparse, and mobility scenarios, the proposed approach is able to present significant results in metrics related to quality-of-service, reliability, and energy efficiency.

## 1. Introduction

The Internet of Things (IoT) is a wide concept that has attracted attention from the research community in recent years [1]. The term IoT can be used to describe a pervasive and ubiquitous network in which devices exchange information between each other without the requirement for human intervention [2]. This network can be used by applications of a wide variety and with varying objectives, such as smart homes and cities [3], industrial automation [4], smart markets [5], and healthcare systems [6,7]. However, at the same time, the IoT has captured the attention of the business world and society. IoT concepts give rise to several technical challenges that limit its broad adoption.

**Figure 1:**
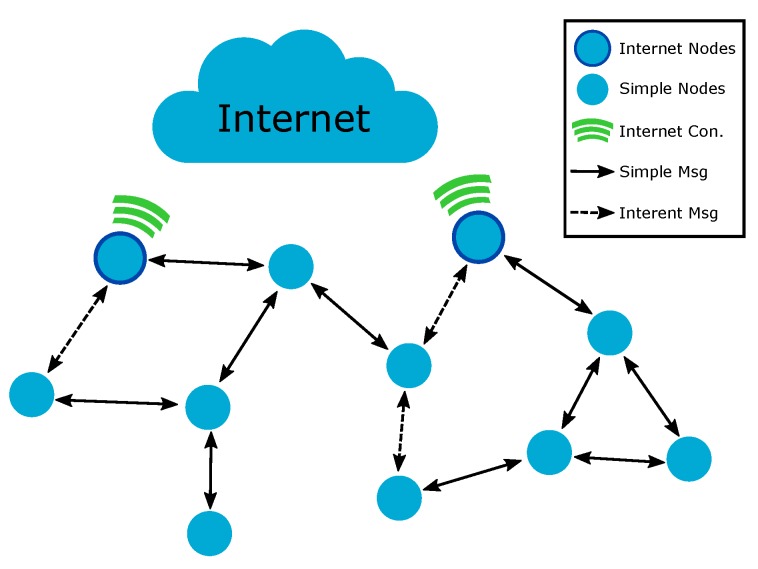
Illustration of an IoT network model with different devices and data message types.

IoT devices form, in general, a low power and lossy network (LLN), composed of many nodes with strong restrictions on memory, processing capacity, and, in some cases, energy. Depending on the application, the nodes in an IoT network can have different hardware capacities and application objectives. In the IoT scenario presented in Figure 1, some network nodes can have a direct Internet connection to send and receive messages from the Internet. In contrast, other nodes from the same network, due to hardware limitations, cannot have a direct Internet connection and require the use of the Internet-connected nodes to access external services. All of these nodes can also exchange information in a local context without the necessity of transmitting data to the Internet. The task of discovering the routes and allowing data messages to be exchanged among nodes is performed by the routing protocol. Thus, in an LLN, the network performance is strongly related to how the routing protocols use the limited hardware resources of the network devices.

In the context of low-power IoT networks, the routing protocols can be grouped into two types based on the route creation principles: proactive and reactive [8]. Proactive protocols begin the creation of routes among the nodes without the prior necessity of data message transmission. The route creation process is, in general, triggered by gateway nodes that have the function of collecting information from other network devices (multipoint-to-point (MP2P) traffic). For this reason, this type of protocol is widely adopted by periodical data collection applications. The main example of a proactive routing protocol for LLNs is the IPv6 Routing Protocol for Low-Power and Lossy Networks (RPL) [9]. In contrast, reactive protocols create routes only when a node intends to send a message to a destination. Thus, the route discovery process is triggered by the node that wants the message. The created routes are maintained in the routing table of the nodes, remaining while in use and removed afterwards. Hence, this type of protocol is indicated for non-periodical traffic application, where any node can send messages to any other (point-to-point (P2P) traffic). The current and most relevant reactive protocol for LLN is LOADng [10]. This work is focused on IoT applications where the traffic pattern is variable, the network devices have different capacities, and the communication among the devices is, mainly, P2P (as exemplified in Figure 1). Thus, considering that reactive protocols are the most appropriate for this type of scenario, LOADng will be used as a basis to the design the proposed solution. The protocol is currently under consideration by the Internet Engineering Task Force (IETF) to become a defined standard. LOADng is in its fifteenth draft and has undergone several modifications since its initial proposal in October 2011 [11]. Thus, the most recent version will be used in this work. A short but comprehensive description of LOADng is presented in Section 3.

Although it uses the most indicated route creation principles in the IoT scenario considered in this study, LOADng can present several problems such as the necessity of previous and static definitions of the nodes responsible for providing the Internet connection to other network devices. Also, the obligation of several routing creation processes to construct on-demand routes to P2P traffic can provoke a high control message overhead. Thus, the main objective of this work is to create an enhancement for LOADng to allow the protocol to better discover and maintain routes for traffic directed to the Internet in IoT networks formed by devices with different capacities. The proposed approach, LOADng-IoT, is composed of three improvements that are able to boost the process of route discovery, reduce the overhead of control messages, and improve the network’s quality-of-service (QoS). In summary, the proposal allows the nodes to find Internet-connected nodes without the prior definition of a gateway. This behavior allows nodes without an Internet connection to forward their data packets to external Internet services with much greater reliability and lower latency. Also, the proposed solution presents a cache system for routes to Internet nodes to reduce the control message overhead required in the process of route discovery. Finally, the solution introduces a new error code that should be used to avoid the loss of data packets to the Internet. Thus, the main contributions of the proposal presented in this work (LOADng-IoT) are as follows:

- LOADng-IoT improves network QoS and reliability by increasing the packet delivery ratio and the reduction of end-to-end latency for the different message types exchanged by nodes in both dense and sparse IoT scenarios.
- It reduces the number of control messages required to construct routes among nodes, contributing to a more efficient network with lower overhead.
- LOADng-IoT reduces the amount of energy required to both build paths and route data messages, making the network more power efficient.
- It dispenses with the use of predefined Internet gateways since the Internet-connected nodes are sought on demand and can change according to their connection availability. This feature also removes the existence of a single point of failure (SPOF) for the connection of IoT devices with external Internet services.
- It presents a flexible solution, whereby parts of the proposal can be adopted according to the hardware capacities of the nodes.

The remainder of this document is organized as follows. Section 2 presents the most important related works on the topic and Section 3 presents an overview of LOADng to provide a clear understanding of the proposed solution. Section 4 gives a detailed description of the proposed LOADng-IoT, while Section 5 describes the used scenario for the performance evaluation, the obtained results, and corresponding discussion concerning the performance of the studied protocols. Finally, Section 6 concludes the paper and offers suggestions for future work.

## 2. Related Work

This work proposes a new enhancement to aid the LOADng route discovery process in IoT scenarios composed of nodes with different capacities and variable message traffic. In the current literature, several studies focus on performance and propose improvements for LOADng. However, to the best of the authors’ knowledge, the current related literature does not propose improvements to the route discovery process of Internet nodes for LOADng in IoT scenarios.

In [12], the authors compared LOADng with RPL in scenarios with different traffic patterns. According to the results obtained, LOADng surpassed RPL in point-to-multipoint (P2MP) and P2P traffic scenarios. However, in the MP2P scenarios, the RPL presented better results. As expected, the proactive features of RPL make it the most appropriate for data collection application scenarios. In contrast, LOADng was able to work better with more generalized traffic but presented a higher delay due to the necessity of realizing the process of route discovery on demand. The performance of LOADng in IoT applications with P2P and MP2P traffic pattern is also studied in [13].

Based on the limited performance of LOADng in MP2P scenarios, Yi and Clausen [14] proposed the LOADng Collection Tree Extension (LOADng-CTP). The proposed enhancement allows the construction of collection trees on-demand to better attend the traffic that flows from the leaf nodes to the root. The proposal was compared with RPL through computational simulations. According to the obtained results, LOADng-CTP obtained delivery ratios, delay, and number of collisions that were very similar to RPL. However, the proposed approach required less control message overhead in all studied scenarios.

A new composite routing metric for LOADng is proposed in [15]. The termed LRRE metric presents an additive approach that merges residual energy (RE), hop count, and number of live routes (LR) in a node. According to the obtained results, the proposed LRRE was able to demonstrate better results when compared to each merged metric used individually. The authors also studied the behavior of the proposed metric in LOADng with a multipath routing adaption. Again, the proposal was able to deliver better results in terms of energy consumption, packet delivery ratio, and load balancing. The impact of the route selection mechanism in the performance of LOADng is widely studied in [16,17].

A multipath improvement for LOADng is also proposed in [18]. The Neighbour-Disjoint Multipath for LOADng (LOADng+NDM) presents a new multipath mechanism adapted for LOADng aiming to improve the network reliability and QoS. The proposed approach creates an initial shortest primary path between a sender and a receiver node. In sequence, it tries to construct a set of backup routes avoiding the nodes that compose the primary path. This behavior seeks to create disjoint routes where backup routes cannot be affected by the failures in the primary path. Simulation and testbed results have exposed the proposed LOADng+NDM overcomes the default LOADng and RPL in simple P2P scenarios. However, in a scenario with multiple P2P messages exchange, the proposed approach have presented higher control overhead and end-to-end latency.

Araújo et al. [19] propose an enhancement routing metric for RPL in IoT environments composed of heterogeneous devices. In the studied scenario, the network was composed of sensor devices, radio frequency tags, and reader-sensor devices. The proposal, based on fuzzy systems, dynamically adapts the routing metrics during the network functioning according to the application requirements. The experimental simulation results showed that the proposed solution was able to overcome the default RPL metrics in terms of energy consumption and packet delivery ratio.

A routing protocol for IoT networks based on the composition of routing metrics is presented in [20]. The Routing by Energy and Link quality (REL) proposes an adaption of Ad-hoc On-demand Distance Vector (AODV) aiming to increase the network reliability and power efficiency. The proposed protocol uses a *weakLinks* mechanism to identify links with low quality across a path. In the route creation process, paths with a high number of *weakLinks* are avoided. The choice of the best route also considers the residual energy of the nodes and hop count. In all the studied scenarios, REL was able to outperform the AODV regarding latency, packet delivery ratio, and network lifetime.

Araújo et al. [21] present a solution based on smartphones to allow the interoperability of IoT devices. The authors propose an architecture in which a smartphone aggregates several communications interfaces to work as a gateway for different technologies. The proposal is software-based and uses only the default implementation of the considered standards. Thus, no improvements were made at the routing layer. A specific testbed was deployed to evaluate the solution in terms of memory, CPU, and energy consumption.

In [22,23], the authors studied solutions for gateway discovery in mobile ad hoc networks (MANETs). The proposals are based on the usage of periodical control messages and consider networks that have mobile nodes or are equipped with an IEEE 802.11 communication interface, which is not the most appropriate for IoT low-power devices. The performance of the solutions was evaluated in terms of packet delivery ratio and end-to-end delay.

Considering the limitations of the current literature and the requirements of IoT low-power networks, this work proposes a new mechanism for LOADng that will allow it to search Internet-connected nodes in a dynamic and on-demand manner. Moreover, the proposed approach can improve normal data traffic among the nodes, enabling the network to become more energy efficient and reliable.

## 3. LOADng Protocol Overview

The LOADng routing protocol proposes a simplification of the AODV [24], a well-known reactive routing protocol based on route discovery using messages of route request and route reply. In LOADng, some aspects are simplified with the aim of reducing the protocol complexity and the amount of computational resources required to execute it. Among the simplifications, it is possible to detach the restriction to the sending of intermediate route reply messages and the avoidance of the use of periodical control messages [16]. Thus, LOADng is specifically designed for networks composed of devices with strong hardware restrictions. Also, it allows the use of different addressing schemes such as IPv6, IPv4, and Rime [25].

The following subsections present an overview of the LOADng protocol. These explanations are necessary to understand the approach proposed in this work.

### 3.1. LOADng Functioning in Brief

LOADng is a reactive routing protocol based on route discovery using route request and route reply messages. Thus, when a node wants to send a data message and the route to the destination is unknown, it should begin a new route discovery process. To this end, the node broadcasts a route request (RREQ) message to search for a route to the desired destination. Each node that receives an RREQ should perform message processing and consider the message to be forwarded. This process continues until the RREQ reaches the sought destination. The destination should then generate a route reply (RREP) message to answer the received RREQ. The RREP is forwarded in unicast to the RREQ originator, constructing a route between the two nodes interested in the message exchange. Finally, the RREP is received by the RREQ originator, which should begin to send data messages using the path created by the route discovery process.

### 3.2. LOAng Control Messages and Information Base

The process of route discovery is performed with the use of control messages inspired by AODV. RREQ messages are always used to request the creation of a route to a destination when a node needs to send a data message and the path to the destination is unknown. RREP messages are used by the destination that receives the RREQ as an answer to the request for route creation. An RREP message may, optionally, require an acknowledgment. In this case, the route reply acknowledgment (RREP_ACK) message is used to answer a received RREP. When a node fails at the moment of data message forwarding, a route error (RERR) message can be used to inform the data message originator of the problem detected. The RERR can also be used when the data message destination is unknown by the intermediate node. Table 1 summarizes the control messages of LOADng and presents its fields with a brief description.

In the process of route discovery, control messages are used in conjunction with an Information Base maintained by each network node. According to the content of the control messages, the Information Set of nodes are fed and updated. The main elements of the Information Base are the following: Routing Set, Blacklisted Neighbor Set, and Pending Acknowledgment Set. The Routing Set is composed by route tuples entries that store data about the neighbor nodes. Based on the Routing Set, a node can verify the existence of a path to a destination or the necessity of starting a new route discovery process. The Blacklisted Neighbor Set is responsible for storing the addresses of nodes with possible communication inconsistencies that make the bidirectional linkage unavailable. The Pending Acknowledgment Set records information about the RREP messages sent with the field ackrequired defined as true. Table 2 presents the fields and the descriptions of the main components of the Information Set.

### 3.3. LOADng Route Discovery

When a node wants to send a data message, it should look for a route to the message destination on its Routing Set. If the path is found, the node should forward the message to its destination through the next hop node. The message forwarding process is described in detail in Section 3.4. However, if the wanted destination is not found, the node should start a new route discovery process.

In the routing discovery process, the node generates a new RREQ message, defining itself as originator and the address of the desired destination in the destination field. It should also set a unique seq-num to the RREQ and define the other message fields. Then, the node should broadcast the generated RREQ to its neighbors.

**Figure 2:**
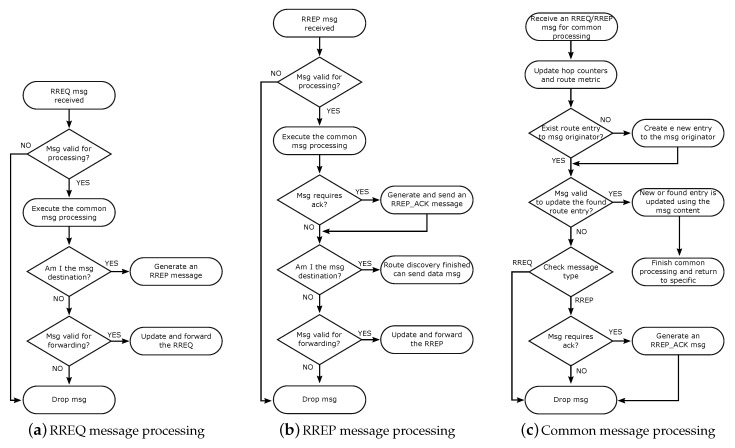
Flowcharts of LOADng RREQ and RREP control messages processing.

Each receiver of the RREQ message should execute its processing according to the flowchart presented in Figure 2a. In the first verification that checks the length of addresses and other details, the message, if considered valid, is subjected to the common processing used both for RREQ and RREP messages. In the common processing (presented in the flowchart in Figure 2c), the node should update the fields of hop-count, hop-limit, and route-metric from the message. In sequence, the node should search for a route entry for the message originator on its Routing Set. If the route is not found, a new route entry for the message originator is created. Then, the created or found route entry is compared with the fields of the received message to verify whether or not the message can be used to update the route entry. If the message is valid, the route entry is updated, the common processing is finished, and the message returns to its specific processing. In contrast, if the message is not used to update the route entry, the node should verify the message type, send an RREP_ACK if required, and drop the message. When the message returns to the specific RREQ processing, the node should check whether it is a message destination. If negative, the node should verify whether the message is valid to be forwarded (checking the hop-count and hop-limit), updating its fields, and forward it using broadcast. Otherwise, if the node is the RREQ destination, it should generate an RREP message to answer the request from the RREQ originator.

The generated RREP message should have the address of the RREQ originator as destination, the address of its originator in the originator field, and a unique seq-num. After being generated, the RREP should be sent in unicast to its destination. Thus, the RREP originator should look for a route entry to the destination on its Route Set and forward the message to the R_next_hop node. Note that the route entry should be found after it has been created by the RREQ message being received. A node that receives the RREP message should perform its processing as described in the flowchart in Figure 2b. After the first validation, the message is submitted to the common message processing (similar to an RREQ message and following the flowchart in Figure 2c). After this processing, the RREP receiver should verify the necessity of generating and sending an RREP_ACK message. In sequence, the node should check whether it is the message destination. If not, the node should consult whether the message is valid to forwarding, verify its Routing Set looking for an entry to the RREP destination and send the message to the R_next_hop node using unicast. Otherwise, if the node is the RREP destination, the route discovery process is completed, and the data message can be sent using the constructed path.

### 3.4. LOADng Data Message Forwarding

In the data message sending process, the node should use the path created in the route discovery process to deliver the data message to its correct destination. Thus, the node consults its Routing Set looking for an entry that matches the message destination. In sequence, the node should forward the message to the next hop of the found route entry. According to the latest LOADng specification, a node should always refresh the valid time of a route entry that it uses. The intermediate node that receives a data message should forward to the next hop of the path based on the information in its Routing Set. This process occurs until the message reaches its final destination. If an intermediate node does not find a route entry that matches the message destination, it should perform a new route discovery process to recover the broken path. If the path recovery does not succeed, the node should generate an RERR message to inform the data message originator of the impossibility of delivering the message successfully.

### 3.5. SmartRREQ Enhancement for LOADng

To reduce the number of control messages exchanged during the route discovery process, the SmartRREQ enhancement was proposed for LOADng [26]. With the use of SmartRREQ, the node should start the route discovery process with an RREQ containing a new smart-rreq flag set as true. Every node that receives a SmartRREQ (RREQ message with smart-rreq true) should perform additional processing in the RREQ message handling. After executing all the initial processing, and after verifying whether the message is valid for forwarding, the node should perform the specific processing of SmartRREQ. Thus, the node checks whether it owns a route entry on its Routing Set to the message destination with R_next_hop that is different from the previous hop of the received SmartRREQ. If this condition is satisfied, the node should transmit the SmartRREQ message in unicast to the next address found. The next hop that receives the SmartRREQ message should perform the same processing until the message reaches the final destination. If a node does not find a route entry to the SmartRREQ destination, the message should be forwarded using broadcast. The destination of a SmartRREQ should answer the request by generating a normal RREP. Hence, the SmartRREQ enhancement can reduce the number of broadcast transmissions, thereby contributing to reducing the control message overhead required to discover a new route and decreasing the network energy consumption.

## 4. Proposed LOADng Enhancement for IoT Networks

This work proposes an enhancement for the LOADng protocol in IoT networks composed of devices with different capabilities. The proposed LOADng-IoT introduces a new route discovery process dedicated to finding devices with the capacity to forward special messages from other nodes. The following subsection explains the IoT applications scenario in which the proposed approach can be applied. In the sequence, LOADng-IoT is fully described, including its features, requirements, and operation.

### 4.1. Considered IoT Applications and Network Model

This work considers an IoT network as presented in Figure 1. The network is composed of simple nodes and Internet-connected nodes, hereafter referred to as INs. The simple nodes represent devices with low capacity, equipped with IEEE 802.15.4, which are unable to realize a direct Internet connection. On the other hand, INs represent devices with high potential, equipped with IEEE 802.15.4 and another communication interface, which are able to provide direct Internet connectivity (such as 4G, Ethernet, or Wi-Fi). All of the network nodes can generate simple messages, which are sent to any network node, and Internet messages, which are directed to external Internet services. The simple messages are locally generated and processed by the nodes. In contrast, Internet messages are created locally and need to reach external services using the Internet. Thus, the INs can directly send Internet messages once they have Internet connectivity. However, simple nodes that generate Internet messages need to find an IN to work as a gateway so that they can then forward their packets. Thus, an IN that receives an Internet message from a simple node should handle and forward the message to the final IP destination using other communication interfaces.

A smart home (SH) IoT application can be used to exemplify the use of the presented network model. In a SH, the smart objects with a low necessity of consulting external Internet services (such as lights, windows, doors, showers, and air-conditioners) can be represented by simple nodes. In contrast, smart objects that require continuous Internet-connection (such as smart meters, smart TVs, smartphones, tablets, and routers) can be represented by INs linked to the Internet using cellular network or optical fiber. Thus, as an example, a smart window can occasionally consult an external Internet service to verify the weather forecast. The smart window, to access the service, should use an IN as its gateway to the Internet. Besides, in a context of a local message exchange, an air-conditioner, when activated, can send a local message to close the smart windows without the necessity of using the Internet to perform this communication.

Current routing solutions can address the presented network model when the simple nodes have been previously configured with a default gateway to forward their Internet messages. Thus, prior knowledge of the nodes with Internet connection capacity is required to then define a gateway for each simple node. This approach, although functional, can give rise to several issues. The obstacles that can occur using this simplistic approach are identified as: (i) high deployment time is required to define the gateway of each simple node; (ii) Internet-connected nodes can be overloaded with Internet messages from simple nodes; (iii) bad deployment can make simple nodes create long paths to their gateways; (iv) simple nodes can become unable to send Internet messages if their gateways lose Internet connectivity.

Based on the exposed constraints, and seeking to better address the requirements of the described IoT network scenario, this work proposes a new enhancement for the LOADng protocol that is able to optimize the route discovery process for INs and improve the network performance. The proposed LOADng-IoT can simplify the discovery of Internet routes, avoiding the necessity of the prior definition of gateways for Internet messages. In addition, the proposal reduces the number of control messages required to construct paths to INs and makes the data message forwarding process more reliable. The following subsections present a detailed description of the proposed LOADng-IoT.

### 4.2. Proposal Overview

The proposed LOADng-IoT is composed of three components: the Internet route discovery process, the Internet Route Cache (IRC) system, and a new error code for RERR messages. The first component is responsible for finding IN nodes without the requirement of previous knowledge of its address in the local network. Thus, a node that wants to send a message to the Internet should start an Internet route discovery process by broadcasting a special RREQ, named RREQ-IoT. The message has the objective of seeking an IN that can be used as a gateway for the RREQ-IoT originator. To reduce the number of broadcast transmissions, an intermediate node that knows a route for an IN can forward the RREQ-IoT message to it using unicast transmission (in the same way as SmartRREQ). When an IN receives an RREQ-IoT message, it should generate a special RREP to answer the request. This message, named the RREP-IoT, is forwarded in unicast via the opposite route created by the RREQ-IoT. Each node that receives an RREP-IoT should create an entry on its Routing Set with the information that the message originator has an Internet connection. When the RREP-IoT reaches its destination, the node should immediately start to send the Internet data messages. The proposed Internet route discovery process of LOADng-IoT is fully described in Section 4.4.

The second component is responsible for storing the Internet routes (routes to Internet-connected nodes) removed from the Routing Set. During the Internet route discovery process, the nodes create entries on the Routing Set to the INs. These entries, which have a valid time, can expire and be removed from the Routing Set when not used. Thus, to reduce the number of transmissions in a new Internet route discovery and to allow the nodes to follow a previously known Internet route, these entries, when removed, have some of its information inserted in a new data structure, the IRC. The IRC should always be consulted when a new Internet route discovery process is started and should, when possible, indicate a previously known Internet route to direct the discovery process. The IRC is optional and should be adopted according to the hardware capacity of the network devices. A complete description of the proposed IRC is presented in Section 4.5.

The third component is a new error code for RERR messages. The INTERNET_CONN_LOST error code should be used to indicate to an Internet data message originator that it was not possible to forward the message to the Internet. Thus, the receiver of the RERR should start a new Internet route discovery process to find a new IN to transmit its Internet messages. The generation, processing, and functioning of the proposed new error code for RERR messages are detailed in Section 4.6.

### 4.3. LOADng-IoT Required Increments and New Features

The proposed LOADng-IoT requires several increments in the existing default LOADng structure. Table 3 shows the fields added to the LOADng to make possible the use of IoT enhancement. Basically, a new field was inserted in both the RREQ and RREP to allow the nodes to identify the messages as IoT and realize special handling. In the Routing Set, a new field was included to enable a route to be identified as an Internet route. Hereafter, in this paper, the term Internet route will be used to refer to a route whose destination is a node with an Internet connection.

In addition to the increments previously presented, the LOADng-IoT also presents several new features required to improve the performance of the studied IoT networks. Table 4 presents these features. The data structure of the proposed IRC is composed of two fields to store the address related to the route entry removed from the Routing Set. The memory required by the IRC structure varies according to the adopted addressing scheme (IPv6, IPv4, or Rime). However, it is important to note that the IRC is optional and could be used just in nodes with slightly greater memory capacity. The option to use or not use the IRC should be configured in each node at the deployment moment using the configuration parameters dedicated to it. In addition, it is possible to define the number of entries in the IRC set according to the memory restriction of the nodes. Considering that, in the Routing Set, the Internet routes may require a valid time that is different from that of common routes, a new parameter was created to allow this feature. Finally, a new error code was created to be used with the LOADng-IoT. The new specific error code allows an Internet node to inform that a received Internet message was not forwarded due to loss of Internet connection. Thus, the Internet data message originator can find a new node to forward its messages.

### 4.4. Internet Route Discovery Process

The Internet route discovery should always be initiated when a node does not find a route entry to an IN on its Routing Set. Thus, the node should create an RREQ with the flag iot set as TRUE (hereafter, this message is named RREQ-IoT). Then, the node defines its own address as the RREQ-IoT originator and destination, and a set new unique seq-num. In the following, the node should consult its IRC (if in use) to verify the existence of a previously known Internet route. At this point, the IRC is considered to be empty, and will be explored in the next subsection. Thus, the node should transmit the generated RREQ-IoT in a broadcast to its neighbors.

**Figure 3:**
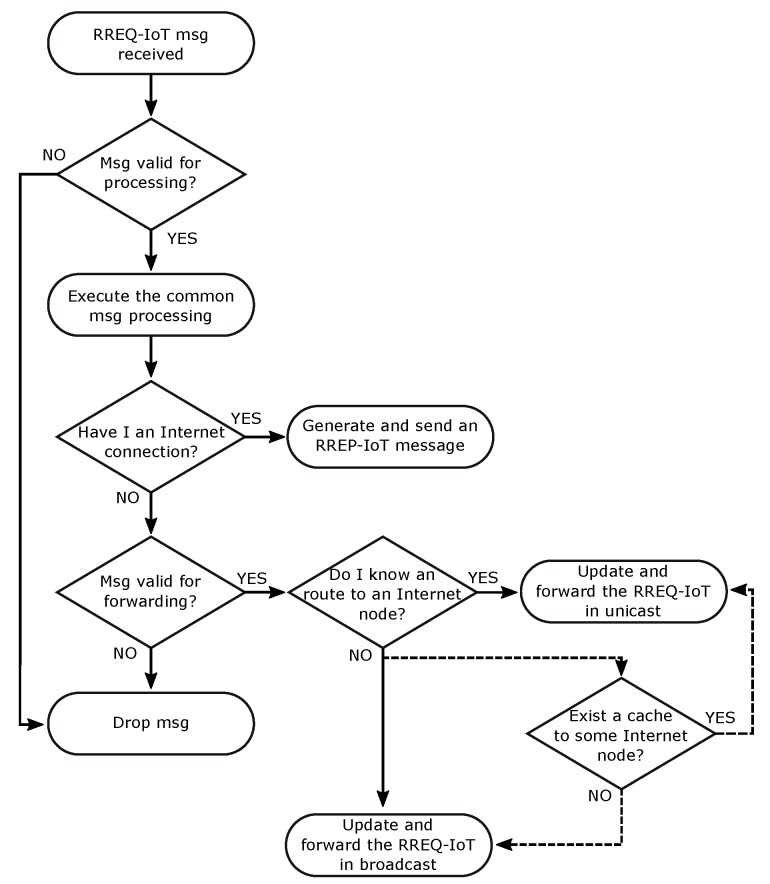
Flowcharts of RREQ-IoT control messages processing. Dotted lines represent the optional flow used when IRC is adopted.

Each node that receives an RREQ-IoT should perform its processing according to the flowchart in Figure 3. Thus, the node first checks whether the message is valid for handling and then conducts the common message processing. The common message processing is done in a similar way as in the standard RREQ message and following the flowchart in Figure 2c. Additionally, during this processing, at the moment a new entry is created in the Routing Set, the node should include the information about the Internet connection status of the message originator. As the RREQ-IoT message originator does not have an Internet connection, the route entry that was created based on the received RREQ-IoT should have the R_Internet_conn set as FALSE. In addition, if the received RREQ-IoT is used to update the information on an existing route entry, the R_Internet_conn should never be changed to false. If the common processing is concluded without the message being dropped, the message handling proceeds to the next step. Thus, in the specific RREQ-IoT processing, the node should check whether it has an active Internet connection (the information on whether or not the node has an Internet connection can be obtained in several ways; this work does not mandate a specific way of doing this. However, as a suggestion, the routing layer can be informed about the Internet connection status through the application layer.). If the node identifies an active Internet connection, it should reply to the RREQ-IoT by generating and transmitting an RREP-IoT message to the request originator. However, if the node does not have an Internet connection, it should check whether the message is valid for forwarding. If true, the node searches for an Internet route on its Routing Set. Thus, if it is found, the node should use a mechanism similar to SmartRREQ to assist the path creation. Hence, the node changes the RREQ-IoT destination to the found R_destination, updates the other message field and sends the message in unicast to the R_next_hop. If more than one Internet route exists in the Routing Set, the node should select the one that best address the used routing metric. However, if an Internet route entry is not found in the Routing Set, the node should use the IRC mechanism, if it is activated. Considering that the node does not use the IRC, it should update the RREQ-IoT message fields and transmit the message in broadcast. Independent of the transmission mode of the RREQ-IoT (unicast or broadcast), the next receiver of the message should perform the process previously described.

**Figure 4:**
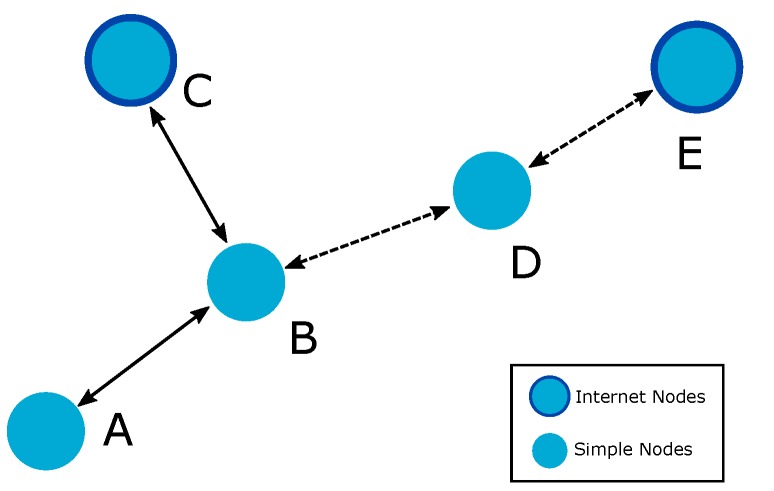
LOADng-IoT Internet route discovery process. Dotted lines represent the linkages allowed but not used.

Please note that unlike the normal route discovery process, the Internet route discovery process searches for any IN rather than a defined destination address. During the RREQ-IoT message processing, the node does not verify whether it is the message destination, but whether it has an active Internet connection. In addition, it is possible that an intermediate node that knows an Internet route will change the RREQ-IoT destination and redirect the Internet route discovery. Figure 4 explains how this may occur. Consider that node *A* needs to send an Internet message and has begun an Internet route discovery process. Node *B* has received the RREQ-IoT from *A* and begun its processing. *B* does not have an Internet connection but finds an Internet route to node *C* on its Routing Set. Thus, *B* changes the RREQ-IoT destination to node *C* and forwards the message in unicast to *C*. Thus, inspired by the SmartRREQ, the Internet route discovery process is optimized to reduce the number of broadcast transmissions, thereby contributing to the reduction of energy consumption.

As previously explained, a node with an Internet connection that receives an RREQ-IoT should generate a reply message to answer the request. Thus, the node generates an RREP-IoT, defines its own address as originator, and the address of RREQ-IoT originator as destination. The message should also have a new unique seq-num. Then, the node transmits the RREP-IoT message in unicast to the next address in the path to the destination.

The node that receives an RREP-IoT should perform its processing, which is very similar to the normal RREP processing, following the flowchart in Figure 2b. Thus, the node verifies whether the message is valid for handling and then executes the common message processing. In the common message processing of an RREP-IoT, all created or updated route entries to the message originator should include the information that R_Internet_conn is TRUE. In addition, the R_valid_time of the Internet route should be, by default, two times greater than the valid time of regular routes. Since only Internet-connected nodes can generate RREP-IoT messages, the intermediate nodes that forward RREP-IoT messages should not be included as R_Internet_conn set as TRUE. Thus, the RREP-IoT message performs the maintenance of routes and creates the path to the Internet nodes. At the end of the common processing, the node can generate an RREP_ACK message, if necessary. In the LOADng-IoT, the RREP_ACK generation, transmission, and processing are equal to the default LOADng. In sequence, the RREP-IoT receiver verifies whether it is the message destination. If false, the node should check whether the message is valid to forward, perform the message update, and transmit it to its destination. Otherwise, if the node is the message destination, the Internet route discovery process is completed, and the node can begin the sending of Internet messages.

In the described Internet route discovery process, all nodes that receive an RREQ-IoT should reply using an RREP-IoT. Thus, it is possible for more than one RREP-IoT to be received by the request originator. This behavior makes the construction of several Internet routes possible, one for each different IN. Hence, a node that intends to send an Internet message should look up its Routing Set and find the best path among those available. The selection of the best Internet route should be made based on the used route metric or the lowest number of hops. The process of Internet message sending and forwarding is described in detail in Section 4.7.

### 4.5. Internet Route Cache for LOADng-IoT

In the course of the network functioning, all route entries of the Routing Set can be removed when the valid time expires. This process occurs to allow the nodes to reduce the memory usage and make the creation of paths to other nodes possible. In the LOADng-IoT, the valid time of the regular routes and the Internet routes can be different and should be adjusted according to the expected traffic in the network. However, considering a scenario where both message types are equality generated, an Internet route tends to be used more by representing a path to all Internet messages. Thus, by default, the authors suggest that the Internet routes have a valid time that is two times greater than regular routes.

Even with a higher valid time, Internet routes that are not used can expire and be removed from the Routing Set of the nodes. Thus, when a node needs to send a new Internet message, the whole Internet route discovery process should be completed again, transmitting several control messages and expending more network resources. To reduce this problem, LOADng-IoT offers an optional improvement that is able to minimize the control message overhead during the construction of Internet routes. This mechanism, the IRC, stores the most relevant information about the last Internet route entries removed from the Routing Set. Then, when it is necessary to perform an Internet route discovery, the node should check its IRC to verify the existence of a previously known Internet route. If positive, the node can direct the Internet route discovery to the destination of the found entry in the IRC.

An entry can be removed from the Routing Set due to valid time expiration or to lack of memory for the insertion of a new entry. Thus, with the use of the IRC, if the removed entry is an Internet route (i.e., R_Internet_conn is TRUE), its R_next_addr and R_dest_addr are used to create a new route cache entry that is inserted in the IRC set. As presented in Section 4.3, the number of route cache entries should be previously defined and should consider the memory limitation of the nodes. The authors suggest that this number be incremented by one for every four possible entries in the routing table. Thus, if the size of the routing table is four, the number of IRC entries should be one; if the size of the routing table is eight, the number of IRC entries should be two. It is also possible to consider the reduction of the number of Routing Set entries to allow the use of the IRC in devices with severe memory restrictions.

The IRC entries do not have valid time and only can be removed by the reception of an RERR message (discussed in Section 4.6). According to the number of route entries defined in the IRC, the oldest entries are removed when a new entry needs to be inserted. In addition, the search for an IRC entry should always get the most recent entry in the set. Thus, the IRC set should work like a stack, where a new entry must be inserted at the start of the set, and the find for an entry should always return the head of the list. When the set is full, the node should to remove the last element in the list and insert the new entry at the beginning.

With the use of the IRC, the nodes should verify its route cache set before starting a new Internet route discovery process. If an entry is found, the node should direct the Internet route discovery process to the destination of the found entry. Thus, the node creates an RREQ-IoT with a destination equal to the found RC_dest_add and sends the message to the RC_next_hop in unicast. The node that receives the RREQ-IoT should realize the message handling normally, as described in Section 4.4. As shown in the flowchart in Figure 3, during the RREQ-IoT processing, a node that does not have an Internet connection should consult its Routing Set to verify the existence of any Internet route. Please note that at this point, as occurs in the normal processing of RREQ-IoT, the destination of the unicasted RREQ-IoT can be changed and the message can be redirected to another node with an Internet connection.

This situation is exemplified in Figure 4. Consider that node *A* needs to send an Internet message and does not find an Internet route on its Routing Set; *A* checks its IRC and finds an entry to the IN *E* with next hop *B*. Thus, *A* generates an RREQ-IoT with a destination equal to the *E* address and unicasts it to node *B* (the found next hop to *E*). *B* receives the RREQ-IoT from *A* and realizes its processing normally. However, *B*, at the moment of checking its Routing Set, finds an Internet route to node *C*. Thus, *B* changes the received RREQ-IoT destination to *C* and forwards the message in unicast. Node *C* then receives the request from *A* and sends a reply offering the required Internet route. This behavior is accepted because the intention of an RREQ-IoT is to reach a route to the Internet, independent of the IN providing the connection. In addition, this redirection of the RREQ-IoT ensures that the Internet route discovery process follows with most recent information (considering that the information provided by the Routing Set is frequently newer than the information provided by the IRC). However, if an Internet route is not found in the Routing Set of the intermediate node, the intermediate node should verify its IRC, change the message destination (if necessary), and then forward the message in unicast to an IN that is able to provide an Internet connection to the RREQ-IoT originator. Finally, whether the IRC set is empty, the node should change the destination address of the RREQ-IoT to the same address as its originator and send the message in broadcast. This process “converts” the RREQ-IoT received in unicast in a normal RREQ-IoT to be broadcasted and the Internet route discovery process can continue until an IN is reached.

The use of the IRC mechanism allows the nodes to reduce the number of control messages required to construct an Internet route. The cache mechanism is used to direct the RREQ-IoTs to a previously known IN. Thus, when adopted, the IRC contributes to the reduction of the number of packet collisions, minimizes energy consumption, and improves network efficiency. As explained, the entries in the IRC set are only removed by lack of memory or the reception of an RERR message. This process is discussed in the description of the new error code proposed in this work in Section 4.6.

### 4.6. Internet Lost Error Code for LOADng-IoT

Due to unexpected situations, the Internet nodes can sometimes lose Internet connection. To avoid these nodes receiving Internet messages when they have no connection, this paper proposes a new error code with the function of advising the neighbor nodes that the Internet connection has been lost. The error code, described as “Internet connection lost” (or *INTERNET_CONN_LOST*), is defined by code 253. The used code number is included in the range of experimental use codes according to the most recent LOADng [10] specifications and can be altered in the future.

During network functioning, when an IN receives an Internet message from another node, it should forward the message to the Internet address destination. If the node detects that the connection has been lost or that it is not possible to realize the forwarding, it should generate an RERR message. The generated RRER message, which has the same structure as the normal RERR presented in Table 1, receives the errorcode 253, the originator as its own node address, and the destination as the originator of the not forwarded Internet message. Notice that, in this case, the field unreachableAddress can be ignored when this error type has not used it. Then, the message is transmitted in unicast to the previous hop of the received Internet message.

**Figure 5:**
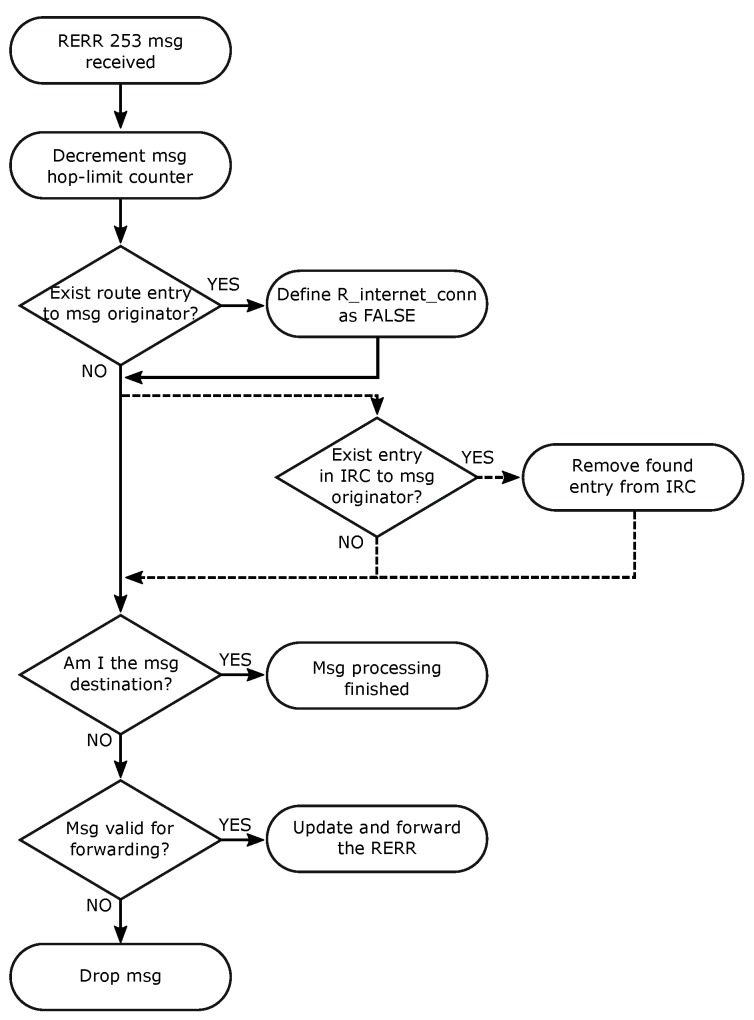
Flowcharts of RERR control messages with code 253 processing. Dotted lines represent the flow used only when IRC is in use.

A node that receives an RRER with code 253 should perform its processing according to the flowchart presented in Figure 5. Thus, the receiver node should initially decrement the hop-limit field. Then, the node should check its Routing Set and, if an entry is found, change the R_Internet_conn to FALSE. If the RERR receiver uses the IRC, it should also verify whether an entry for the RERR originator exists on its IRC set. If an entry is found, the node should remove it. Notice that the route updated to R_Internet_conn false is not removed from the table and is not included in the IRC set. This procedure avoids the node trying to start a new Internet route discovery from using information about the node with the connection lost. In sequence, the node verifies if itself is the message destination. If true, the RERR process is completed and the message is not forwarded. Otherwise, the node should ascertain whether the message is valid for forwarding, update the message fields, and send the message to its destination.

An RERR message with code 253 can also be generated by an intermediate node in the path to the destination of an Internet message. An intermediate node that receives an Internet message and detects that the message destination does not have an active Internet connection should start a new Internet route discovery process to find a new Internet path. If the path is not successfully created, the RERR message is generated to the Internet message originator.

The use of the proposed error code allows the network to reduce the number of Internet messages sent to a node without an Internet connection. Thus, the nodes are able to find alternative Internet routes when they receive an RERR message with code 253. As a benefit, the number of packets lost can be reduced, which contributes to improving the network efficiency and reliability.

### 4.7. LOADng-IoT Data Message Forwarding

In the network operation with the use of the proposed LOADng-IoT, the nodes that intend to send data messages should verify its Routing Set and execute the route discovery process, if necessary. After constructing the routes, simple messages and Internet messages can be sent to its destinations through the next hop node. In the case of simples messages, as the LOADng constructs only one path to each destination, it is not possible to compare the best route for sending (the best path selection is made during the process of route discovery). However, to send an Internet message, the node should to select the best path based on the selected routing metric since the Internet route discovery process can create several routes to different INs. Thus, it is possible to choose the best path among those available. The nodes that receive a data message (both simple and Internet) should consult its Routing Set to find the next hop to the destination node. This process should continue until the data message is delivered.

Independent of the message type, it is possible for a broken route to occur during the message forwarding process. In this case, the intermediate node that was not able to forward the message detects the broken path, queues the data message, and starts a new route discovery process according to the message type. If the message is simple, the node should begin a normal route discovery using the standard LOADng procedure. However, if the message is directed to the Internet, the node should start an Internet route discovery following the process described in Section 4.4. Thus, the node should consult its IRC set (if enabled) and send an RREQ-IoT unicast or broadcast following whole the process. In both cases, if the route is constructed successfully, the queued data message is forwarded normally. Otherwise, if it is not possible to reconstruct the path, the node should generate an RERR according to the data message type and send it to the data message originator.

## 5. Performance Evaluation and Results Analysis

This section presents the performance assessment realized to evaluate the behavior of the proposed solution. The Cooja simulator/emulator, which is a part of the Contiki O.S. [27], was used for this purpose. Although the use computational simulations cannot precisely represent the behavior of a network in the real world, it can allow a fair comparison among routing protocols since it makes the reproduction of an identical environment possible for all studied proposals [25]. In addition, because it works as an emulator, Cooja permits the replication of the hardware conditions of real nodes such as Tmote Sky, Zolertia Z1, and others. Thus, each emulated node represents the identical hardware conditions of real devices in terms of memory usage and processing capacity.

The proposed LOADng-IoT was compared with the most recent version of LOADng and LOADng-SmartRREQ. The objective was to analyze the behavior of the proposed solution with the other proposals in different scenarios. Thus, situations were created with three topology organizations: grid sparse, random dense, and mobility dense. For all the topologies, the number of nodes in the network changed from 16 to 64. This quantity was chosen because it can represent the majority of the existing small-scale IoT application scenarios, mainly in smart homes. The following itemization presents more details on the used grid, random, and mobility topologies.

- Grid Sparse Scenarios: the network nodes were organized in linear grids of *nxn* nodes. The simulated area grew together with the number of nodes. Thus, a fixed network density was maintained where the nodes had between two and four neighbors.
- Random Dense Scenarios: the different quantity of nodes was randomly deployed in an area of 200 square meters just once. Thus, the random deployments were the same for all compared proposals. The simulated area was the same for the different quantities of nodes. Hence, the network density grew with the increase in the number of nodes.
- Mobility Dense Scenarios: the nodes were deployed with the same positions of random dense scenarios in an area of 200 square meters. However, the nodes with an Internet connection was able to move in the whole area of the studied environment.

For all scenarios and the different quantities of nodes, the simulation time was 600 s. In the application, all network nodes generated and sent data messages in variable intervals of between 10 and 15 s. The minimum data message interval was defined as 10 to avoid the nodes being overloaded with several data messages while they were still realizing a route discovery process. This measure was required because the nodes do not implement a significant buffer for data messages in the routing layer. Thus, all data messages generated inside a route discovery process are lost. The data message generation should be able to address the requirement of Equation (1):

(1) 2∗(1+RREQ_RETRIES)∗NET_TRANSVERSAL_TIME

where RREQ_RETRIES is the maximum number of route discovery retries and NET_TRANSVERSAL_TIME is the expected time to a control message traversing the whole network. The generated data messages were simple messages or Internet messages, as explained in Section 4.1. Both messages were generated randomly with a chance of 50%. Thus, the simulated scenarios created a network traffic pattern that merged P2P (sending simple messages from a local node to another local node) and MP2P (sending Internet message from local nodes without an Internet connection to a local node with an Internet connection).

In the simulated scenarios, the number of INs grew according to the number of nodes in the network, representing around 10% of network devices. To simulate an environment where the IN could, periodically, lose its Internet connection, these nodes had a random Internet connection time of between 60 and 90 s. After this time, the connection remained lost for a random time of between 0 and 60 s. In sequence, the connections were once again reestablished with a new random time of between 60 and 90 s. The most critical parameters of the simulation are presented in Table 5. The parameters of the nodes used in the simulation environment are presented in Table 6. Notice that INs simulate an additional communication interface for the Internet connection. In the mobility scenario, the mobile nodes, which are the INs, move according to the random waypoint mobility model [28]. The BonnMotion 3.0.1 [29] tool was used to generate the movement of the mobile nodes. Table 7 presents the parameters of mobility used in this study. All studied approaches were based on LOADng and used the same settings presented in Table 8. However, the proposed approach (LOADng-IoT) used two more parameters, presented in Table 9.

The following subsections show the obtained results for the metrics of the packet delivery ratio (PDR), the energy spent per delivered data bit (AES), the control message overhead per delivered data message (CMO), and the percentage of packets with low latency (PLL). For all studied metrics, the simulations were executed 30 times, and the results presented a confidence interval of 95%.

### 5.1. Packet Delivery Ratio

The PDR metric represents the number of data messages that were successfully delivered to the destination node. Thus, a high PDR represents an efficient network that is able to deliver the generated data messages with high reliability. This metric is constantly affected by the quality of the links among the nodes, the radio interference provoked by neighbor devices, and collisions with other data and control messages. In this paper, the Internet messages delivered for a node without an Internet connection were considered lost. The PDR value of the network was obtained according to Equation (2):

(2) $$PDR=\frac{\sum_{i=1}^{N}Pr_{i}}{\sum_{i=1}^{N}Ps_{i}}$$

where *N* is the number of nodes in the network, $Pr_{i}$ is the number of data packets received for each node *i*, and $Ps_{i}$ is the number of data packets sent by each node *i*.

**Figure 6:**
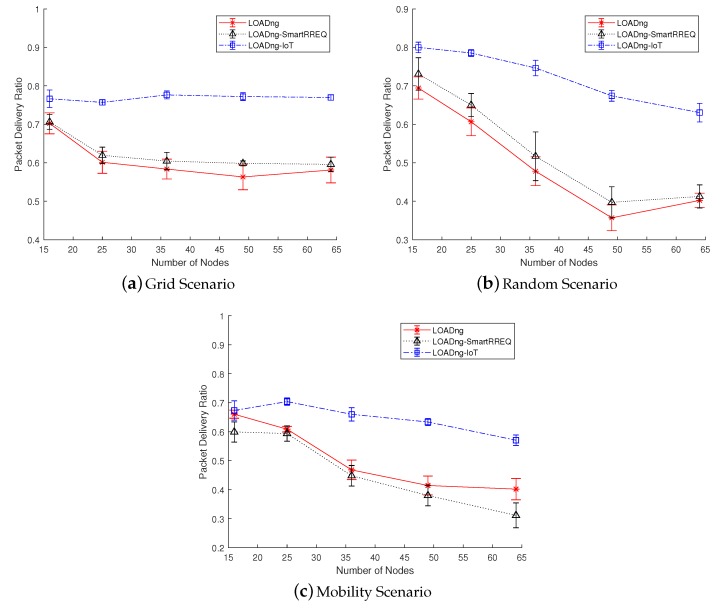
Packet delivery ratio in function of number of nodes.

Figure 6 presents the results obtained for the PDR metric. In all studied scenarios, the proposed LOADng-IoT obtained better results when compared with the other approaches. In the grid scenario, where the network was sparse and the quantity of neighbor nodes was constant, the proposed solution presented satisfactory results, demonstrating its scalability and reaching a PDR of between 75% and 80%. In contrast, the other approaches decreased its performance with the increase in the number of nodes. The reason for this behavior was the dependency on a fixed Internet-connected node to send the Internet messages. In addition, networks using LOADng and LOADng-SmartRREQ were unable to detect an IN’s loss of Internet connection. Thus, the Internet messages were continuously forwarded and lost due to the incapacity of the IN to route them to the Internet. With the use of the proposed approach, the INs were able to use the new error code to inform the Internet message originator when its Internet connection was lost. Thus, the message originator was able to start a new process of Internet route discovery to find a new gateway to forward its messages to the Internet. In the random and mobility scenarios, where the network was dense, the same behavior was perceived. However, the obtained PDR values were lower for all the studied networks, mainly in the mobility scenario. The reduction of the network performance is already expected when the network density grows. As the number of nodes is increased in a fixed area, the probability of packet collisions grows, provoking a high packet loss. However, the proposed solution was able to obtain better results (in some cases, 40% better than default LOADng). The movement of INs also contributes to the packet loss since paths previously constructed can rapidly become unavailable due to the mobility of the nodes. In this case, the nodes that detect a route broken should use RERR messages to inform the data originator about the forwarding incapacity. This behavior provokes the necessity of a new route discovery implying in the transmission of control messages and, consequently, increasing the energy consumption. However, as the mechanism used by LOADng-IoT to find routes requires fewer control messages when compared with the other approaches, the losses provoked by Internet-connected nodes movement are reduced. Hence, with the lower necessity of radio transmissions, the probabilities of packet collisions and message loss are decreased.

### 5.2. Average Energy Spent per Delivered Data Bit

The average energy spent per delivered data bit metric represents the amount of energy spent by the network to successfully delivery each data bit to its destination. Thus, the less energy spent to deliver the data successfully, the higher the power efficiency of the network. The results obtained by this metric are affected by the energy consumption of the nodes and the packet delivery ratio. The metric is computed using Equation (3):

(3) $$AES=\frac{\sum_{i=1}^{N}Ec_{i}}{\sum_{i=1}^{N}Pr_{i}*M_{length}}$$

where *N* is the number of nodes in the network, $Ec_{i}$ is the total energy consumed (in millijoules) by each node *i*, $M_{length}$ is the length of the data message in bits, and $Pr_{i}$ is the number data packets received by each node *i*.

**Figure 7:**
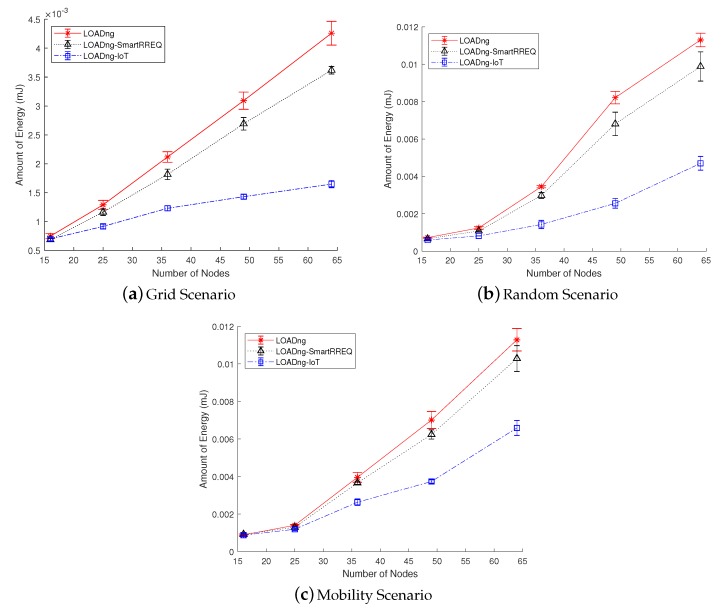
Average energy spent per delivered data bit in function of number of nodes.

Figure 7 shows the results obtained for the AES metric. In all studied network scenarios with more than 25 nodes, the proposed LOADng-IoT was able to obtain better results when compared with the other studied approaches. Considering the grid sparse scenario with 64 nodes, LOADng-IoT outperformed LOADng by ∼168%, and LOADng-SmartRREQ by ∼125%. In the mobility scenario, also with 64 nodes, the proposed approach is able to give a performance ∼73% better when compared with LOADng. In general, the reduction in the number of control messages used in the route discovery process allowed the proposed protocol to use fewer radio transmissions, provoking the energy consumption decrement. The improved process of route discovery for Internet nodes also enabled the protocol to find closer destinations to forward the Internet messages. Thus, the data messages delivered to the Internet-connected nodes required fewer transmissions, contributing to the reduction of energy consumption. Less transmission also implies a lower probability of packet loss caused by radio interference or packet collisions. Hence, by reducing the energy consumption and reaching a high packet delivery ratio, LOADng-IoT was able to spend less energy to deliver each data bit, exposing a high power efficiency in comparison with LOADng and LOADng-SmartRREQ.

### 5.3. Control Message Overhead per Delivered Data Message

The control message overhead per delivered data message (CMO) metric shows the number of control message transmissions required to deliver each data message successfully. As in the previously presented metric, the results of the CMO are directly related to the packet delivery efficiency. Although control messages are not used during the forwarding of data packets, it is possible to count the overhead generated by the nodes to construct the path used to deliver the data messages. Thus, calculating the ratio between the quantity of control message transmission used to discover the routes and the number of data packets delivered, it is possible to obtain the mean of control message transmissions required to deliver each data message. Thus, this metric is calculated according to Equation (4):

(4) $$CMO=\frac{\sum_{i=1}^{N}CMt_{i}}{\sum_{i=1}^{N}Pr_{i}}$$

where *N* is the number of nodes in the network, $CMt_{i}$ is the total number of control message transmissions performed by each node *i*, and $Pr_{i}$ is the number of data packets received by each node *i*.

**Figure 8:**
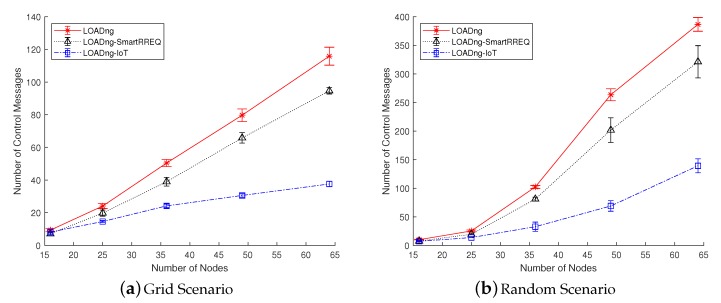

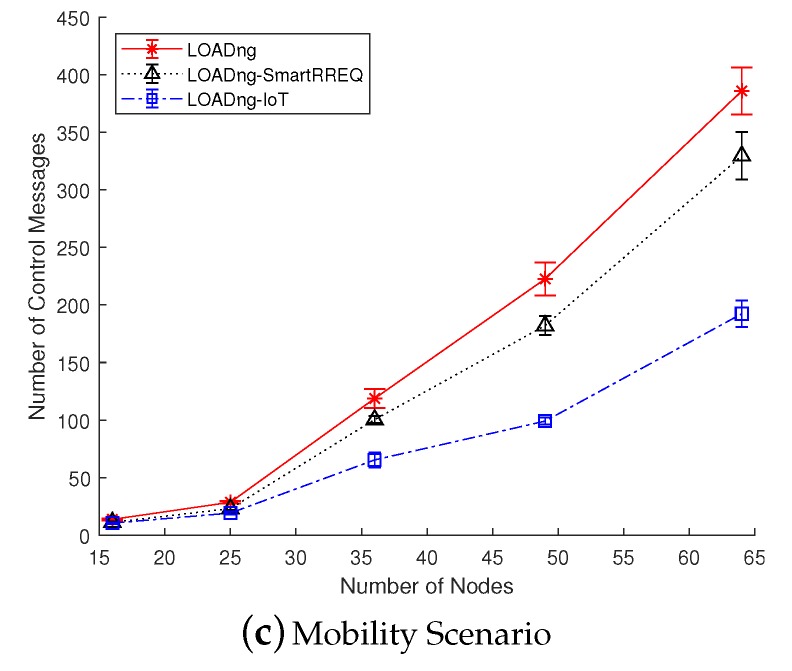
Control message overhead per delivered data message in function of number of nodes.

The results obtained for the CMO metric are presented in Figure 8. In all studied scenarios, the proposed solution was able to obtain better results concerning the compared approaches. The main advantage of using LOADng-IoT is the reduction in the number of control message transmissions during the route discovery process. The mechanism of Internet route discovery, which was inspired by LOADng-SmartRREQ, has reduced the number of control message broadcasts avoiding the nodes outside the already known route received and processed control messages irrelevant to them. In addition, the facility provided by the IRC has allowed the nodes to improve the IN discovery, since the route discovery is directed to a previously known IN using unicast transmissions. Notice that the mobility of the INs was not able to affect the benefits of IRC. The capacity of LOADng-IoT converting a unicasted RREQ into a normal RREQ allows the node to discover a path to an Internet-connected node even if IRC has directed the RREQ to an IN no more available. In the results from all studied scenarios, LOADng-IoT outperforms LOADng and LOADng-SmartRREQ being able to, in some cases, reduce by three times the number of control message transmissions required to deliver one data message. It is also important to note that, in the random and mobility scenarios, the control overhead is about two times greater than in the grid sparse scenario. In addition, in general, the number of control message transmissions is elevated in relation to the number of delivered messages. This behavior is justified by the configurations adopted in the network and protocol parameters. The data throughput is low and the valid times of the routes are short, meaning that a high number of route creation processes is required by the nodes. These parameters were purposely adjusted in this way to permit a better analysis of the behavior of the studied protocols.

### 5.4. Percentage of Packets with Low Latency

The metric of the percentage of packets with low latency exposes the percentage of data packets delivered with a latency that is considered low and acceptable for low-power devices in IoT applications. Several aspects can affect the metric results; among these, it is possible to cite: the length of the path constructed during the route discovery process; the quality of links among the nodes; the radio duty cycle frequency; the medium access control protocol; the density of nodes, etc. The PLL metric is computed according to Equation (5):

(5) $$PLL=\frac{\sum_{i=1}^{N}lat\left(Pr_{i}\right)<L_{th}}{\sum_{i=1}^{N}Pr_{i}}$$

where *N* is the number of nodes in the network, $Pr_{i}$ is the number of packets received by each node *i*, lat() is a function that returns the latency of each packet received Pr, and $L_{th}$ is the latency threshold of a data packet be considered delivered with low latency. In this work, the $L_{th}$ was defined in 500 milliseconds.

**Figure 9:**
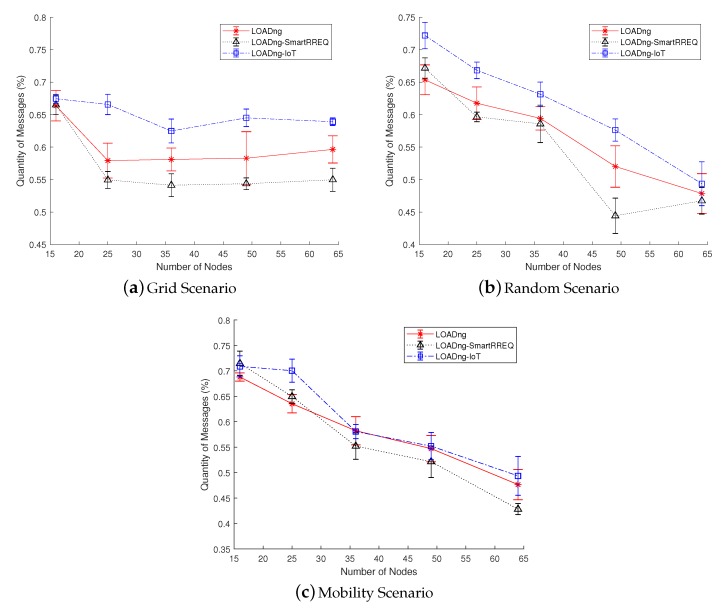
Percentage of packets with low latency in function of number of nodes.

Figure 9 shows the results obtained for the metric of PLL. According to the results, the proposed LOADng-IoT was able to overcome the other compared solutions in the majority of studied network configurations. In the grid sparse network, except for the network with 16 nodes, the studied approaches were able to maintain almost constant results. Thus, LOADng-IoT delivered approximately 65% of the data packets with a latency lower than 0.5 s. This result outperformed the default LOADng by ∼8% and LOADng-SmartRREQ by ∼14%. With the use of LOADng-IoT, the nodes do not need to use a predefined gateway to forward Internet messages. Thus, the process of Internet route discovery was able to find closer nodes, constructing shorter paths and reducing the number of hops required to send an Internet message. In the dense random scenarios, the proposed LOADng-IoT was also superior to the other studied proposals. In the mobility scenario, the performance of all studied approaches was almost the same, except for the network with 25 nodes, where LOADng-IoT presented slight better results. However, it was perceived that the performance of all approaches decreased with the increase in network density. This behavior is already expected, considering that the latency tends to be increased when several nodes try to use the same frequency spectrum in a common region. The MAC protocols tend to spend more time sending the messages due to the high number of devices accessing the wireless medium [30].

## 6. Conclusions and Future Works

This work presents a new improvement to the LOADng routing protocol in IoT scenarios when the network devices have different capacities and use different message types. The proposal was compared with the default implementation of LOADng and LOADng-SmartRREQ through simulations using COOJA. Four different metrics were studied to expose the network performance in terms of reliability, QoS, and power efficiency. For all the considered metrics, LOADng-IoT demonstrated better performance for sparse, dense, and mobile networks. These significant results were obtained due to the set of improvements provided by the proposed approach.

Unlike other approaches, the proposed LOADng-IoT does not require a previous definition of gateways and, hence, it can find the most appropriated Internet node to forward messages. This feature provides a self-adaption capacity to the network nodes and reduces the necessity of human intervention in both network deployment and execution. Optionally, LOADng-IoT allows the use of a cache system dedicated to storing Internet routes, enabling the nodes to direct the route discovery to INs that anteriorly had been used as gateways. These approaches together permits LOADng-IoT to reduce the control message overhead required to find the Internet routes, contributing to the reduction of power consumption and improving the packet delivery ratio. Finally, this work proposed a new error code that makes it possible for the Internet nodes to advise the other network nodes about its temporary Internet connection loss. Thus, devices that intend to send Internet messages can find new gateways to forward messages, increasing the chances of successful delivery. It was also noted that LOADng-IoT performance was, in general, less variable when compared with the other studied proposals. This behavior is justified because LOADng-IoT requires fewer radio transmissions to perform the data packet delivery and route discovery. Thus, LOADng-IoT is less affected by the interferences and collision that commonly makes the network performance unstable. In conclusion, the authors deduce that, together, all of the proposed mechanisms that comprise LOADng-IoT provide a new significant enhancement for IoT networks, allowing them to attain better QoS, efficiency, and reliability.

For future work, the authors suggest that experiments in real IoT environments should be conducted to test the results obtained using computational simulation. Moreover, the source code from the proposed solution should be improved, documented, and disseminated to the scientific community.

## Figures and Tables

**Table 1:** LOADng Control Messages.

RREQ Message	
**Field**	**Description**
addr-length	Defines the length of the addresses used by originator and destination nodes
seq-num	Indicates the sequence number that uniquely identifies each message generated by the originator node
metric-type	Determines the type of metric used by the message originator node
route-metric	Defines the value of the route metric of the path
hop-count	Indicates the number of hops that the message has traversed
hop-limit	Indicates the maximum number of times that a message can be forwarded
originator	Specifies the address of the message originator
destination	Specifies the address of the message destination
**RREP Message** (contains the same fields as RREQ plus the above)	
**Field**	**Description**
ackrequired	Indicates the necessity of generating an RREP_ACK when be received
**RREP_ACK Message**	
**Field**	**Description**
addr-length	Defines the length of the addresses used by originator and destination nodes
seq-num	Indicates the sequence number of the RREP messages that was triggered the generation of the RREP_ACK
destination	Specifies the address of the message destination
**RERR Message**	
**Field**	**Description**
addr-length	Defines the length of the addresses used by originator and destination nodes
errorcode	Indicates the error code of the message
unreachableAddress	Specifies the address of the node unable to be reached
originator	Specifies the address of the message originator
destination	Specifies the address of the message destination
hop-limit	Indicates the maximum number of times that a message can be forwarded

**Table 2:** LOADng Information Base.

Routing Set	
**Field**	**Description**
R_dest_addr	Indicates the address of the route destination
R_next_addr	Indicates the address of the next hop in the path to the route destination
R_metric	Specifies the value of the metric computed for the path to the destination nodes
R_metric_type	Determines the route metric used to compute the metric value
R_hop_count	Specifies the number of hops to the route destination
R_seq_num	Indicates the sequence number of the control message used to generate the entry in the set
R_bidirectional	Indicates if the message is bidirectional
R_local_iface_addr	Specifies the communication interface used to reach the route destination (used only when a node has more than one interface)
R_valid_time	Specifies the length of time the entry is considered valid in the set
**Blacklisted Neighbor Set**	
**Field**	**Description**
B_neighbor_address	Indicates the address of the blacklisted neighbor
B_valid_time	Specifies the length of time the entry is considered valid in the set
**Pending Acknowledgement Set**	
**Field**	**Description**
P_next_hop	Indicates the address of the node that the RREP was sent
P_originator	Indicates the address of the RREP originator
P_seq_num	Defines the sequence number of the sent RREP
P_ack_received	Determines whether the pending RREP_ACK was received
P_ack_timeout	Specifies the length of time the entry is considered valid in the set

**Table 3:** LOADng-IoT required increments in to LOADng structure.

RREQ and RREP	
**Field**	**Description**
iot	Indicates that the RREQ or RREP message is *-IoT; when used in RREQ, indicates that the originator is searching for an Internet node; when used in RREP, indicates that the originator has an Internet connection.
**Routing Set**	
**Field**	**Description**
R_Internet_conn	Indicates the address of the blacklisted neighbor

**Table 4:** LOADng-IoT new features.

Internet Route Cache	
**Field**	**Description**
RC_dest_addr	Specifies the address of the destination removed from the Routing Set
RC_next_hop_addr	Specifies the address of the next hop to reach the destination removed from the Routing Set
**Configuration Parameters**	
**Field**	**Description**
R_INTERNET_HOLD_TIME	Defines a valid time to an Internet route entry in the Routing Set
USE_INTERNET_ROUTE_CACHE	Specifies if the node uses the IRC mechanism
NUM_ROUTE_CACHE_ENTRIES	Indicates the number of entries supported in the IRC
**Error Code**	
**Field**	**Description**
INTERNET_CONN_LOST	Indicates the impossibility of forwarding a data message to the Internet due to lost; the error code is 253.

**Table 5:** Parameters of Simulation.

Parameter	Value
Network Area	150∼280 m^2^
Number of Nodes	16∼64
Num. of Internet-connected Nodes	2∼6
Simulation Time	600 s
Radio Environment	Unit Disk Graph Model (UDGM)-Distance Loss
Transmission Range	50 m
Interference Range	50 m
TX and RX Chance	90%
Data Message Frequency	10 s∼15 s
Data Message Length	512 bits
Traffic Pattern	P2P and MP2P
Medium Access Control (MAC) Protocol	Carrier Sense Multiple Access (CSMA)
Radio Duty Cycle (RDC) Protocol	ContikiMAC
Check Channel Rate (CCR)	16 Hz

**Table 6:** Nodes Parameters.

Parameter	Value
Mote Type	Tmote Sky
Radio	CC2420
Max. Transmission Power	31 dBm
Supply Voltage	3.6 v
TX Current Consumption	21.0 mW
RX Current Consumption	23.0 mW
Low Power Mode (LPM) Current Consumption	1.2 mW
CPU Current Consumption	2.4 mW

**Table 7:** Mobility Parameters.

Parameter	Value
Mobility Model	Random Waypoint
Mobility Area	200 m^2^
Max. Speed	3 m/s
Min. Speed	1 m/s
Max. Pause Time	60 s
Min. Pause Time	0 s

**Table 8:** Parameters of LOADng.

Parameter	Value
NET_TRANSVERAL_TIME	2
RREQ_RETRIES	1
RREQ_MIN_INTERVAL	2
R_HOLD_TIME	60
MAX_DIST	65,535
B_HOLD_TIME	4
MAX_HOP_LIMIT	255
RREQ_MAX_JITTER	1
RREP_ACK_REQUIRED	FALSE
USE_BIDIRECTIONAL_LINK_ONLY	FALSE
RREP_ACK_TIMEOUT	2
MAX_HOP_COUNT	255
NUM_RS_ENTRIES	8
NUM_BLACKLIST_ENTRIES	16
METRIC_TYPE	HOP_COUNT

**Table 9:** Parameters of LOADng-IoT.

Parameter	Value
R_INTERNET_HOLD_TIME	120
USE_INTERNET_ROUTE_CAHCE	TRUE
NUM_ROUTE_CACHE_ENTRIES	2
